# Editorial: From molecular changes to behaviour: exploring neuropsychiatric disorders

**DOI:** 10.3389/fpsyt.2026.1893034

**Published:** 2026-06-22

**Authors:** Bruno Manadas, Cátia Santa, Diana Rodrigues

**Affiliations:** 1 CNC – Center for Neurosciences and Cell Biology, and CIBB – Centre for Innovative Biomedicine and Biotechnology, University of Coimbra, Coimbra, Portugal; 2 BioMed X Institute, Heidelberg, Germany; 3 Division of Physiological Genomics, BioMedical Center, Faculty of Medicine, Ludwig-Maximilians-Universität München, Planegg-Martinsried, Germany

**Keywords:** animal models, biomarkers, endocannabinoid system, epigenetics, mitochondrial dysfunction, neurodevelopmental disorders, neuropsychiatric disorders, non-pharmacological interventions

## Introduction

Neuropsychiatric and neurodevelopmental disorders sit at the intersection of biology and behavior, where genetic, molecular, cellular, and environmental processes converge to shape phenotypes that include schizophrenia, mood disorders, suicidality, neurodevelopmental conditions, and dementia-related psychosis, among others. Despite the impressive accumulation of genome-wide, omics, neuroimaging, and electrophysiological data over the past two decades, the field continues to grapple with a foundational question: how do specific molecular and cellular alterations translate into the diverse, often overlapping, behavioral manifestations observed in patients? Closing this molecular-to-behavior gap is essential for developing objective biomarkers, mechanistically grounded interventions, and, ultimately, a more precise psychiatry.

This Research Topic, “From Molecular Changes to Behavior: Exploring Neuropsychiatric Disorders,” brings together ten contributions that address this challenge from complementary perspectives. The articles span basic, preclinical, and clinical research and cover biochemical alterations and neurotransmitter dynamics, omics-derived biomarkers, cellular and mitochondrial pathology, epigenetic regulation, electrophysiological signatures, animal models, and non-pharmacological interventions. Three threads run through the collection: (i) the value of multidimensional, integrative approaches linking behavior to biochemical, anatomical, and functional readouts; (ii) the translational potential of well-characterized mechanisms as biomarkers and therapeutic targets; and (iii) careful engagement with the biological heterogeneity that defines these disorders.

## Molecular signatures and emerging biomarkers

The first set of articles addresses the search for objective, peripherally accessible biomarkers of psychiatric risk and disease. Lv et al. use two-sample Mendelian randomization with data from the Psychiatric Genomics Consortium to assess causality between IgG N-glycosylation traits and psychiatric outcomes. They report a protective effect of genetically predicted IGP7 across schizophrenia, major depressive disorder (MDD), and bipolar disorder, along with disorder-specific associations involving IGP21, IGP22, IGP34, and IGP57. These findings position IgG glycomics as a candidate window into the inflammation–psychiatry interface and as an avenue for noninvasive risk prediction.

Yang et al. combine RNA sequencing of peripheral blood mononuclear cells with an integrated machine-learning pipeline (LASSO, SVM-RFE, and Random Forest) to identify peripheral biomarkers of adolescent MDD. Their work converges on a three-gene panel (SLC4A1, IGF1, MMP9) that yields an area under the curve of 0.867 and simultaneously implicates an unexpected coexistence of hypoxic compensation and neurotrophic/remodeling failure in early-onset depression. The result is a striking illustration of how unbiased transcriptomics can refine diagnosis while reshaping pathophysiological hypotheses.

Malekpour et al. address suicidality, one of psychiatry’s most urgent yet least mechanistically understood phenotypes. Their systematic review and bioinformatic synthesis of human case–control studies converge on three recurrently dysregulated microRNAs (miR-30a, miR-30e, and miR-218) whose brain-expressed targets implicate FoxO and Rap1 signaling, long-term depression, and dopaminergic synapse function. Together, these findings delineate actionable biomarker and therapeutic target candidates for a phenotype that has resisted single-marker approaches.

Kesebir and Demirer complement these omics-based efforts with an electrophysiological approach, integrating advanced information-theoretic measures of EEG dynamics (entropy-doubling, Ruzsa distance) with peripheral lactate levels in patients with mania-predominant bipolar disorder. High-beta amplitude entropy emerges as the strongest and most consistent correlate of lactate, supporting a model of metabolic–oxidative dysregulation that bridges brain dynamics and mitochondrial bioenergetics and offering a quantitative biomarker framework at the interface of psychiatry and biomedical engineering.

## Mechanisms of vulnerability and disease

A second cluster of articles examines the mechanistic processes linking environmental and molecular insults to disease. Shkarina et al. review how chronic stress remodels the epigenome during critical developmental windows, altering the expression of genes that govern neural maturation and function. By cataloging the epigenetic modifiers and target genes most consistently implicated, they make the case for precision-medicine approaches operating at the epigenomic level, both as diagnostic strata and as therapeutic entry points.

Rao et al. synthesize an evolving body of literature on the type-1 cannabinoid receptor (CB1R) as a shared substrate of psychosis in schizophrenia and Alzheimer’s disease. They argue that CB1R functions as both a “friend” and a “foe”. Its loss, whether driven by adolescent high-potency cannabis exposure, anandamide-driven internalization in first-episode psychosis, or age-related decline in Alzheimer’s disease, is associated with psychotic symptomatology. Low-dose partial agonism appears to alleviate it. The paper weaves together cannabinoid pharmacology, epigenetic regulation of CNR1, and clinical findings into a coherent translational narrative.

Lai et al. shift the focus to the subcellular scale, showing that the SENP3/FIS1 axis drives mitochondrial fragmentation in the prefrontal cortex of mice subjected to chronic unpredictable mild stress (CUMS) and that electroacupuncture at GV20/GV29 reverses this fragmentation alongside depression-like behavior. This study is an instructive example of how a non-pharmacological intervention can be mechanistically linked to a defined molecular pathway, with clear implications for circuit- and organelle-targeted therapeutics.

## From models to interventions

A final cluster looks ahead, asking how new model systems and interventions can accelerate translation. Elgueta-Reyes et al. make the case for the underexploited utility of Drosophila melanogaster in studying the negative symptoms of schizophrenia: anhedonia, social withdrawal, and apathy, which have remained refractory in mammalian models. Genetic dissection of fly homologs (dysb1, Rim, Neuroligins), combined with refined behavioral paradigms, offers a high-throughput route to mechanistic and therapeutic discovery for symptoms with limited pharmacological options.

Zhang et al. revisit the endocannabinoid system from a different vantage point: as a mediator of the antidepressant effects of physical exercise. They present evidence that moderate aerobic exercise increases anandamide and 2-AG, engages CB1R signaling, and reshapes reward and stress-resilience circuits through neuroplasticity, hippocampal neurogenesis, and neuroimmune regulation. Read alongside Rao et al., this contribution highlights how the same endogenous signaling system can mediate vulnerability in one context and resilience in another: a vivid illustration of biological complexity that rewards mechanism-aware, dose- and context-sensitive interventions.

## Concluding remarks

Taken together, these ten contributions illustrate that progress in neuropsychiatric research depends on holding several scales in view at once: peripheral biomarker discovery alongside studies of synaptic and mitochondrial biology; animal and cellular models alongside human clinical and post-mortem data; and pharmacological alongside lifestyle and somatic interventions. They also remind us that the same molecular system (be it the endocannabinoid axis, mitochondrial dynamics, or the epigenome) may carry opposing prognostic and therapeutic implications, depending on the developmental window, disorder, and dose. We thank the authors for their thoughtful submissions, the reviewers for their rigorous critique, and the editorial staff of Frontiers in Psychiatry for their support throughout this Research Topic. We hope this Research Topic encourages further multidimensional studies that continue to translate molecular changes into mechanistic understanding of behavior and, in time, into better lives for the people who live with these conditions.

